# Treatment Outcomes and Associated Factors among Tuberculosis Patients from Selected Rural Eastern Cape Hospitals: An Ambidirectional Study

**DOI:** 10.3390/tropicalmed8060315

**Published:** 2023-06-09

**Authors:** Lindiwe M. Faye, Mojisola C. Hosu, Joshua Iruedo, Sandeep Vasaikar, Kolisa A. Nokoyo, Urgent Tsuro, Teke Apalata

**Affiliations:** 1 Department of Laboratory Medicine and Pathology, Walter Sisulu University and National Health Laboratory Services (NHLS), Private Bag X5117, Mthatha 5099, South Africa; mojisolahosu@gmail.com (M.C.H.); sandeepvasaikar@yahoo.com (S.V.); ruffinapalata@gmail.com (T.A.); 2 Department of Family Medicine, Walter Sisulu University, Private Bag X5117, Mthatha 5099, South Africa; joshuairuedol@gmail.com; 3 Wits School of Public Health, 27 St Andrew Road, Parktown, Johannesburg 2193, South Africa; sikhuthali@gmail.com; 4 Department of Public Health, Walter Sisulu University, Private Bag X5117, Mthatha 5099, South Africa; tsurourgent@gmail.com

**Keywords:** treatment outcomes, DR-TB, MDR-TB, TB-HIV co-infection, treatment success rate

## Abstract

An essential metric for determining the efficacy of tuberculosis (TB) control programs is the evaluation of TB treatment outcomes; this study was conducted to investigate treatment outcomes and associated factors among tuberculosis patients in rural areas of Eastern Cape, South Africa. Assessing treatment outcomes is fundamental to facilitating the End TB Strategy’s set target. Clinic records from 457 patients with DR-TB were examined for data collection while 101 patients were followed up prospectively. Data were analyzed using Stata version 17.0. The odds ratio and 95% confidence interval were calculated to check the association between variables. *p* ≤ 0.05 was considered statistically significant. Of the 427 participants, 65.8% had successful treatment whilst 34.2% had unsuccessful TB treatment. A total of 61.2% and 39% of the HIV-positive and HIV-negative participants had a successful TB treatment whilst 66% and 34% of both HIV-negative and positive participants had unsuccessful TB treatment. From the 101 patients that were followed up, smokers took longer to have treatment outcomes compared to non-smokers. In the study with HIV/TB co-infection, men predominated. HIV and tuberculosis co-infection made therapy difficult with unfavorable effects on TB management. The treatment success rate (65.8%) was lower than the WHO threshold standard with a high proportion of patients being lost to the follow up. The co-infection of tuberculosis and HIV resulted in undesirable treatment outcomes. Strengthening TB surveillance and control is recommended.

## 1. Introduction

Worldwide, tuberculosis (TB) is a significant public health menace and South Africa (SA) is among the top 30 nations grappling with a high burden of TB. Identified nations contributed 87% of the estimated incident TB cases in 2018 [1,2]. Comparably to many other African countries, SA is laden with the triple burden of TB, TB-HIV co-morbidity, and drug-resistant TB. Drug-resistant (DR) TB is a result of resistance to at least one first-line anti-TB medication [3,4]. A major worry for TB control strategies is the rising incidence of multidrug-resistant (MDR) TB and extensively drug-resistant tuberculosis (XDR-TB) [5].

The interconnectivity of TB epidemiology with social and economic conditions has made its prevention and control a daunting task to achieve [6]. Notably, previous studies found that socioeconomic conditions such as undernutrition, alcohol and substance abuse, smoking, and unemployment contributed to an increased risk of TB, recurrent TB even after the completion of treatment and poor treatment adherence and outcomes [7,8,9,10]. Co-morbidities with cancer, HIV and diabetes mellitus and adverse treatment reactions from second-line drugs (SLID) also facilitated undesirable outcomes in MDR-TB [9,10].

Treatment success rate (TSR) is a critical factor to the global End TB strategy. For this factor, a 90% rate was determined as a standard for all countries to actualize [11]. With the 76% national TSR in South Africa, the country still falls short of the standard set by WHO, the global health body [1]. The low TSR obtainable in the continent of Africa can be attributed in part to the loss of participants to follow ups and discontinued treatment due to death [12]. Though interventions such as the directly observed short-course therapy-plus (DOTS-plus) have been reported to improve TSR in MDR-TB patients, interruption of the treatment regimen still occurs in some patients. [13,14,15,16]. The interruption of treatment in MDR-TB patients is a predictor for the emergence of further drug resistance strains, such as XDR-TB or pre-XDR-TB and total drug resistant-TB (TDR-TB) [17,18,19], and results in a 3–4-fold increase in the risk of a poor treatment outcome [13].

Evaluating TB treatment outcomes is important to assess the efficacy of treatment interventions, improve systemic inadequacies, develop strategies, and make informed decisions on the efficient management of DR-TB [12,20]. Research on the profile and management of tuberculosis treatment outcomes and related factors in the study area is scarce. Consequently, the findings of this study are critical for the study areas in order to lessen the impact and identify predictors of good treatment outcomes. The study assessed TB treatment outcomes and associated factors among TB patients in selected Eastern Cape hospitals in South Africa for the period covering January 2018 to December 2019.

## 2. Materials and Methods

### 2.1. Study Design, Setting and Participants

This was an ambidirectional study in which a review of medical records was conducted to assess the treatment outcome of TB patients and the associated factors from TB patients of rural Eastern Cape who were initiated for TB treatment from January 2018 to December 2019 and few patients were followed up from the initiation of treatment to the treatment outcome stage. The healthcare facilities (5 hospitals and 1 referral hospital in total) of the study were selected from districts of Eastern Cape Province, South Africa, that are serviced by or under the demarcation of Nelson Mandela Academic Hospital National Health Laboratory Services TB Laboratory. The Eastern Cape province is the third biggest province out of nine provinces in South Africa and has a total population of approximately 7 million people [21].

### 2.2. Data Collection

The South African National Tuberculosis and Control Program (NTCP) report format was used by the researcher and trained research assistants to collect data from the medical records of tuberculosis patients who began treatment between 2018 and 2019. The medical records with missing information were excluded. There were 457 patients who were enrolled in the study. Of these, 101 patients were randomly selected based on convenience, i.e., geographic accessibility to the study site at baseline visits, and followed up until the end of their treatment. The data collected included socio-demographics, clinical data, and treatment outcomes.

### 2.3. Variables of the Study

- The dependent variable was a tuberculosis treatment outcome which was a successful tuberculosis treatment outcome (cured and treatment complete) and an unsuccessful treatment outcome. Unsuccessful treatment outcomes included failure, loss to follow up, and death.
- The independent variables were age, gender, HIV status, pattern of *Mycobacterium tuberculosis* resistance, type of resistance, previous drug history, and period between the start and end of the treatment
- The primary exposures were the period between the beginning and end of the study, the resistance type, the nature of TB drug resistance, and previous drug history. HIV status was included as an a priori.

### 2.4. Operational Definition of Treatment Outcomes

The South African National Tuberculosis and Control Program (NTCP) guidelines based on standard WHO definitions were used to define treatment variables [22].

(i) Cured: this category included patients who finished treatment with negative bacteriology results at the end of the treatment or with a negative sputum smear on two occasions at the end of treatment.
(ii) Completed: these were patients with documented treatment completion but without bacteriology results at the end of the treatment.
(iii) Treatment failed: patients whose sputum smear remained positive at five months despite the correct intake of medication.
(iv) Defaulted treatment: these were patients who had been on treatment for at least 4 weeks and who interrupted their treatment for two consecutive months or more after registration, and still had a positive smear with active TB.
(v) Died: patients who died from any cause during the course of TB treatment.
(vi) Transfer-out patients: patients whose treatment results were unknown due to transfer to another health facility or another district.
(vii) Successful: the sum of cases that were ‘cured’ and those who ‘completed’ treatment.
(viii) New TB patient: a TB case who had not previously been treated or treated for less than a month for TB, was subsequently diagnosed and started the current treatment.

### 2.5. Data Processing

The treatment outcome was combined and recorded into two groups which were successful treatment outcomes (cured and treatment completed) and unsuccessful treatment outcomes (died, defaulted, transferred out, treatment failure, and lost to follow-up).

### 2.6. Statistical Analysis

All statistical analyses were performed using STATA Version 17.0 SE (Stata Corporation, College Station, TX, USA) [23]. A *p*-value of <0.05 was considered as statistically significant. The baseline characteristics of the study patients were reported according to the 5 primary exposure variables, with age and gender being used to determine confounding and/or effect modification characteristics when all other variables were adjusted for. Categorical variables were summarized as frequencies and percentages. Bivariable and multivariable logistic regression analyses were used to determine the relationship between dependent and independent variables. A bivariate analysis was performed to identify factors associated with the treatment outcome of patients with MDR-TB. A multi-variable logistic regression analysis was employed to determine the independent predictors of the treatment outcomes of patients with MDR-TB. The results of the logistic regression are expressed as crude and adjusted odds ratios. Differences in proportions for categorical variables was evaluated using the chi-square tests and means with standard deviations were used for continuous variables. Binary logistic regression was performed to examine the associations between the treatment outcomes and number of days of treatment, previous drug history, HIV status, drug resistance type, and nature of drug resistance together with gender and categorized and uncategorized age. The associations are reported as odds ratios with 95% confidence intervals. All potential confounders were included in the final model, then stepwise regression (forward selection and backward elimination) using a flexible *p*-value of 0.1 was performed so that statistically significant variables were not excluded. A goodness of fit was performed in the final model to establish the model strength and whether or not it could be used in the final analysis. Sensitivity and specificity tests were conducted, and 84% of the participant were correctly classified whilst 10 of the participants were identified as outliers.

## 3. Results

### 3.1. Characteristics of the Study Participants

Of the 457 participants on TB treatment whose data were captured, 30 (6.6%) participants were excluded due to duplicate data. Of these, 427 (93.4%) patients had complete data and were therefore included in the study. A total of 281 (65.8%) had a successful treatment whilst 146 (34.2%) had unsuccessful TB treatment. The age distribution of the patients ranged from 15–90 years, with a mean age of 46.63 ± 16.24 years. HIV/TB coinfection was observed in 82 (9.20%) of the patients. Over half of the patients, 151 (53.7%), were in the 21–40 age bracket in the successful treatment group with a mean age of 38 years and minimum age of 15 years. With the unsuccessful treatment group, half of the patients, 73 (50%), were in the 21–40 age bracket with a mean age of 36 years and a minimum age of 15 years as well. The average number of days of the period between the start and end of treatment in the successful group was 326.5 days with a minimum of 95 days while the unsuccessful group had an average of 186 days with 82.6 days as a minimum. An average of 130 males and females had successful treatment and 59 had unsuccessful treatment. Regarding previous drug history, more than half, 160 (56.9%), of the patients with successful treatment, never had any exposure (new group) to a TB treatment regime. A total of 130 (46%) and 151 (53%) of the patients in the successful treatment category had mono- and poly-resistance, respectively, while 71 (48.6%) and 75 (51%) patients displayed mono- and poly-resistance, respectively, in the unsuccessful treatment category. In the successful treatment group, 131 (46.6%) and 130 (46.3%) participants were confirmed to have rifampicin resistance (RR) and MDR-TB, respectively. A total of 172 (61.2%) and 109 (39%) of the HIV-positive and HIV-negative participants, respectively, had a successful TB treatment (Table 1). Once the age was categorized further, most participants for both successful and unsuccessful treatment belonged to the age category group of 40 years in which 151 (53.7%) and 73 (50%) participants were recorded.

### 3.2. Association of the Treatment Outcome with the Period between the Start and End of Treatment and Previous Drug History

Of the 427 participants, 281 (65.8%) had a successful TB treatment (classified as either cured or treatment completed) whilst 146 (34.2%) had an unsuccessful treatment (classified as loss to follow up, failed, transferred, died, and still on treatment). This translated to a treatment success rate (TSR) of 65.8%. There was no reference group in the study. The odds ratio for treatment days (the period between the start and end of treatment) indicates that every unit increase in the number of days between the start and the end of TB treatment is associated with a 1.6% decrease in the odds of having a successful TB treatment. The odds ratio for drug history (previous drug history) indicates that every unit increase in the previous drug history is associated with an 84% increase in the odds of having successful TB treatment. This is confirmed by the *p*-values that are less than 0.05 and the 95% confidence intervals that do not exceed zero or one (Table 2).

### 3.3. Factors Associated with Treatment Outcome

With a certain type of TB drug resistance, an increase valued of resistance to rifampicin and isoniazid is associated with 9% and 54.5% in the odds of having an unsuccessful TB treatment, respectively, with the *p*-value of 0.012 being statistically significant as shown in Table 1. When added to the logistic regression model with drug history and treatment days, the drug resistance becomes slightly statistically significant with a *p*-value of 0.064 (Table 3a). The addition of gender and HIV status in the model shows that gender has confounding effects on drug resistance.

In the age category, ages below 5 and over 90 were omitted from the analysis. Being under 21 and exactly 21 years of age indicates that each increase in age within this category is associated with increasing odds of successful TB treatment whilst in the age categories 40, 55, and 75, each unit increase in age is associated with decreasing odds of successfully completing the TB treatment. When categorized, not all the age categories were statistically significant, but the overall age had slight statistical significance, with a *p*-value of 0.083 when the stepwise regression *p*-values were applied at 0.1. Being either male or female increased the odds of successfully completing the TB treatment but this variable is also not statistically significant. There was no statistical significance of whether the participant had HIV or not, but the odds of having a successful TB treatment increased with having a negative HIV status.

Overall, HIV status and gender had no statistical significance in both the earlier model and the stepwise regression models of the analysis with the *p*-values of 0.186 and 0.118, respectively; however, the HIV status will be included as a priori as TB infection and HIV in South Africa are treated concurrently.

The final model selected, the stepwise regression model (similar in both forward selection and backward elimination) confirms what the other models presented regarding the association of the treatment outcome with the number of days in treatment, previous drug history, age of the participant, and finally the type of drug resistance (Table 3b). The resistance type, whether mono- or poly-resistant, and the gender had no statistical significance on whether the participants would successfully or unsuccessfully complete the TB treatment.

### 3.4. Socio-Demographics of TB Patients That Were Followed Up from Baseline Visits to Treatment Outcome Visits

**Figure 1:**
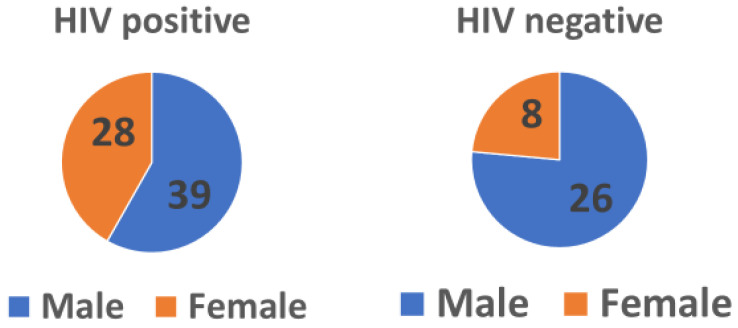
Relationship between patient’s gender and HIV status.

The number of patients enrolled in the study was 101. Of these, 65 (64.4%) were males and 36 (35.6%) were females. Among all these patients, 67 (66.3%) were HIV-positive, while 34 (33.7%) were HIV-negative. About half of the participants, 52 (51.5%), belonged to the economically active age group (Table 4). Gender is a risk factor associated with treatment outcomes with a *p*-value of 0.012, and HIV status had a marginal *p*-value (0.05). More males were co-infected with HIV-TB (Figure 1) than females.

### 3.5. Treatment Outcomes of TB Patients That Were Followed Up from Baseline Visits to Treatment Outcome Visits

At 24 months of follow up, a total of 42 (41.6%) and 32 (31.7%) of the patients completed their treatment and were cured, respectively, giving a successful treatment outcome of 73.3% (n = 74). Thus, the composite TSR for patients included in this study was 73.3%. An unsuccessful treatment outcome was observed in 27 (26.7%) patients. Of these, 10 (9.9%) patients died of the disease at 24 months of the follow-up period. In total, eight (7.9%) were lost to follow-up (LTFU) and transferred out to other treatment centers each. Treatment failure occurred in one patient (1.0%).

### 3.6. Factors Associated with Treatment Outcomes of TB Patients That Were Followed Up from Baseline Visits to Treatment Outcome Visits

**Figure 2:**
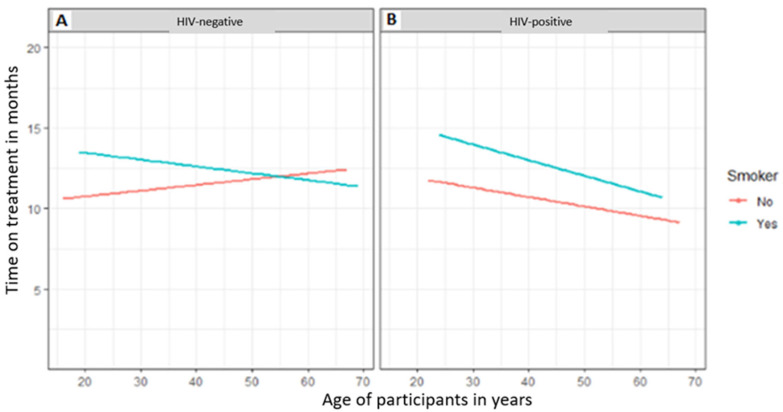
Comparison of patient smoking history to period of treatment in relation to HIV status and age. (**A**) HIV-negative participants; (**B**) HIV-positive participants.

The relationship between the time on treatment and the age of the study participants according to HIV status based on whether they smoke tobacco or not is indicated in Figure 2. According to Figure 2A, HIV-negative participants who smoked had a higher time of treatment initially, but as age increased the time on treatment decreased while non-smokers had a lower time on treatment at a younger age, which increased with age. As depicted in Figure 2B, HIV-positive participants who smoked tobacco had a longer time on treatment at a younger age, which decreased with age; on the other hand, non-smokers had a reduced time on treatment at a younger age. HIV-positive young smoking patients had prolonged treatment periods compared to HIV-positive old smoking patients (Figure 2B).

**Figure 3:**
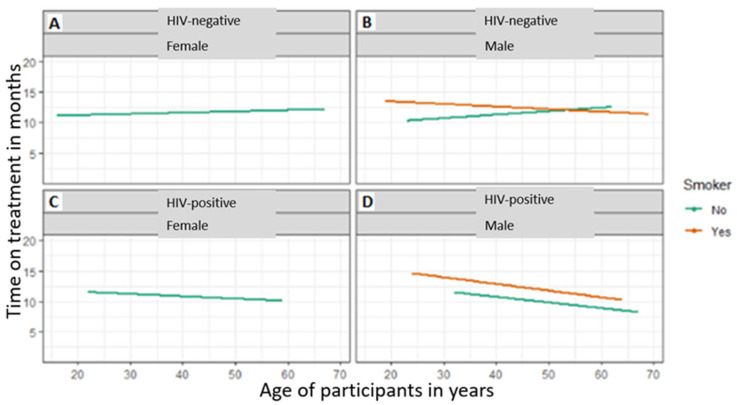
Comparison of patient’s gender to period of treatment in relation to HIV status. (**A**) HIV-negative females, (**B**) HIV-negative males (**C**), HIV-positive females and (**D**) HIV-positive males.

All female patients were non-smokers as highlighted by Figure 3A,C. HIV-negative males’ period of treatment outcomes decreased with an increase in age (Figure 3B). Both non-smoking females and males had an increased period of treatment outcomes with an increase in age. HIV-positive male smokers took longer under the treatment compared to non-smokers (Figure 3D). Both the smoking and non-smoking HIV-negative or -positive period of treatment outcomes decreased with an increase in age (Figure 3A,B).

**Figure 4:**
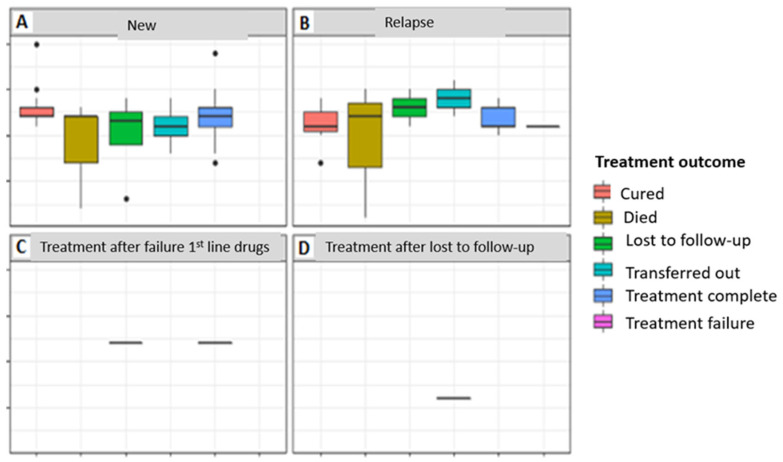
**A**–**D** Comparison of treatment outcomes with the period of treatment in relation to patient category of HIV-negative and -positive patients: (**A**) New patient category; (**B**). Relapsed patient category; (**C**). Patients who were re-treated after failure of 1st-line drugs; (**D**). Patients who were re-treated after loss to follow up.

In the patient category, the most common was new patients, which accounted for 65.3% (n = 66). Other categories included retreatment relapse, treatment failure after first-line drugs (retreatment after failure), and treatment after loss to follow-up (retreatment after LTFU). The number of HIV-negative patients was n = 25, and that of HIV-positive patients was n = 41. Treatment failure was observed in a relapsed patient while transferred out and dead patients were in the new and relapsed categories, with more deaths observed in the category of relapsed patients (Figure 4).

**Figure 5:**
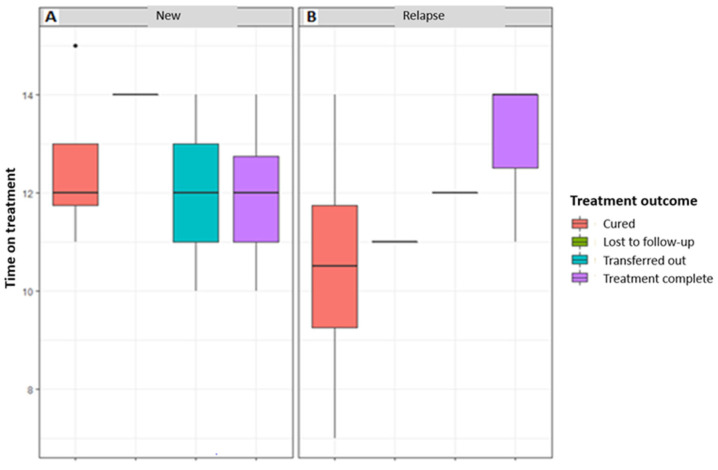
Comparison of treatment outcome to the period of treatment in relation to the patient category of HIV-negative patients: (**A**) treatment outcome in new patients; (**B**) treatment outcome in relapsed patients.

All new TB patients had the same median treatment outcome time, which was approximately 12 months, except for those lost to the follow up, for whom this was approximately 14 months (Figure 5A). Patients that were cured of TB had the smallest median treatment outcome time, but with a wider interquartile range, while those who completed treatment had the longest duration of treatment (Figure 5B).

**Figure 6:**
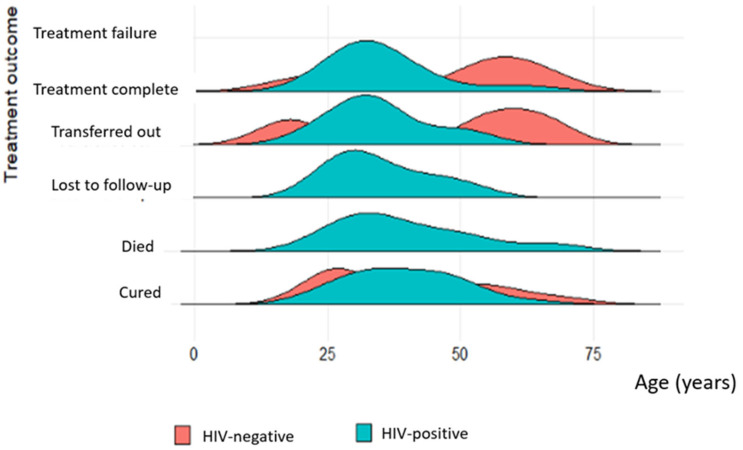
The distribution of age over treatment outcomes based on HIV status.

In HIV-negative patients, there were younger and older people who had treatment failure, completed treatment, and were cured compared to HIV-positive patients. The economically active age group had more patients in all categories of treatment outcomes (Figure 6).

**Figure 7:**
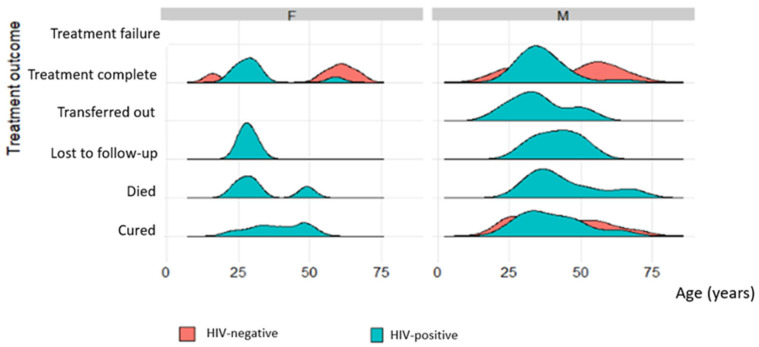
The distribution of age according to gender over treatment outcome based on HIV status.

In both genders, there were no HIV-negative patients that died or were lost to the follow up. There were more HIV-negative patients that were cured and had completed treatment (in both genders) in the younger and older age groups compared to the economically active age group. Only HIV-positive patients in the economically active age group were transferred out (Figure 7).

Only a small number of patients (n = 11) showed heteroresistance. Of these, six had successful treatment outcomes and five did not. Only one patient had mixed infections, and that patient was transferred out, indicating that the patient’s therapy was ineffective.

## 4. Discussion

Evaluating the treatment outcome of tuberculosis and identifying the associated factors should be an integral part of tuberculosis treatment both at district and national levels. Millions of TB fatalities each year have been alleviated and prevented thanks to better TB diagnosis and effective treatment [24]. However, there are a number of obstacles that sub-Saharan African TB treatment structures must overcome in order to be as highly effective as possible. As a result, these issues lead to unsatisfactory treatment outcomes. The study was conceptualized to assess and understand the determinants of both successful and unsuccessful treatment outcomes in TB patients who received treatment at selected healthcare facilities of selected districts of Eastern Cape. The study focused more on HIV-TB coinfection and TB treatment outcomes.

Treatment success rate (TSR) is a helpful measure for evaluating the efficacy of the tuberculosis control campaign. The implication of a low TSR is that TB-infected patients may not be receiving adequate treatment and stand the risk of developing drug-resistant TB which could serve as a potential reservoir for the transmission of MDR-TB [25]. Although the overall treatment success rate of 65.8% obtained in this study was lower compared to the 86% global average achieved in 2020 [26] and the 90% target advocated by WHO, this was higher than that of 57.4% in Kwazulu-Natal Province [1] but lower than that of 80% from Gauteng Province in South Africa [27]. The TSR of other sub-Saharan African countries was recorded as the following: 95% in Mozambique [28], 73.1% in Zambia [29], 73% in Botswana [19], and 61.1% in Zimbabwe [30]. The intermediate TB success rate within our study points to the underperforming and weakened TB programs in a resource-constrained area. The disparity in treatment success might be a result of the research’s sample size, geographic location, study period, study population, or study setting. Furthermore, variations in how TB treatment regimens are applied could also account for this disparity [24,31].

The study findings revealed a higher percentage of TB cases amongst the age groups of 21–40 and 41–55. This is corroborated by other studies conducted in Kwazulu-Natal, South Africa, as well as in Ghana and Mozambique, in the African continent [1,24,28]. This implies that those most severely affected fall within the productive age group which can have a negative impact on the economy if not controlled. TB treatment care must be given utmost priority. Apart from the social mobility associated with this productive age group, HIV co-infection is so common among people in this age bracket in South Africa, and this might be another contributing factor [1]. Although previous evidence suggested age as a marker in determining TB treatment outcomes [32], no significant association was found between all the age groups and unsuccessful treatment outcomes in this study. Our study revealed that gender and the type of drug resistance had no association with treatment outcomes.

There was no statistical significance of whether the participant had HIV or not, but the odds of having a successful TB treatment increased with having a negative HIV status. Tuberculosis is a common opportunistic infection in people living with HIV/AIDS; these infections, termed the “deadly duo”, are considered major public health problems in sub-Saharan Africa [33,34]. The co-infection of tuberculosis and HIV challenges treatment, resulting in undesirable outcomes of TB treatment. The prevalence of HIV co-infection (63.4%) in this study was much higher than that reported in another Eastern Cape study (57.1%) [35]. According to the 2022 Global TB Report, 710,200 of the 10.6 million new TB cases in 2021 had concurrent HIV infection and were concentrated in countries that make up the WHO African region, exceeding 50% in parts of southern Africa [26]. Several factors, such as drug interactions, overlapping drug toxicities, the exacerbation of side effects, dwindling TB treatment adherence due to high pill liability, immune reconstitution inflammatory syndrome, poor absorption of anti-TB medications such as rifampicin and ethambutol, which can result in drug resistance and treatment failure, make managing HIV infections in people with TB more challenging [34,36]. Consistent with our results on high prevalence of TB/HIV co-infection are the findings of previous studies conducted in Mozambique and Zimbabwe [28,30]. Inadequate HIV therapy during the TB occurrence and increased drug burden for the co-infected patient are potential causes of this. Moreover, poor absorption of anti-TB medications, a high pill burden, and the inadequate disease information often associated with HIV-positive tuberculosis patients lead to a much worse prognosis after TB treatment [28,37]. Additionally, the interaction between the infections may accelerate the progression of one or the other. The significance of TB screening and preventative therapy in people living with HIV (PLHIV) cannot be over-emphasized as a result of the high TB/HIV prevalence and higher risk of unfavorable TB treatment outcomes observed in this population [28]. Integrated TB/HIV care has significantly improved over the past ten years as a result of the country’s high dual TB and HIV burdens. Nevertheless, more needs to be carried out for those with dual infections, particularly if ART has not yet been established [35].

In the part of our study that related to patients followed-up from the initiation of treatment, the male gender dominated the study with HIV/TB co-infection. TB was more prevalent in the productive age group both among HIV-positive and HIV-negative TB patients as similarly observed in other studies and in consonance with global epidemiological results [37,38,39,40]. The disproportionately high frequency of TB in men has previously been linked to access issues and delays in seeking care. Prior research has linked access issues and delays in seeking care to the disproportionately high prevalence of tuberculosis among men [41]. The engagement of males in more social activities than women are engaged in also increases their risk of developing secondary infections [42]. Men’s risk of TB acquisition may be further increased by undiagnosed and untreated HIV co-infection as well as missed opportunities for screening TB within HIV care [43,44]. There was no association between gender and treatment outcomes, but female patients were more likely to have successful treatment outcomes compared to male patients.

The treatment success rate (73.3%) observed in the current cohort was in line with the success rates reported by a retrospective cohort study in Sierra Leone (73%) [38], a study conducted in Zambia (73.1%) [29], one conducted in Uganda (71.8%) [45], and one conducted in China (69.6%) [40]. However, it was relatively higher than the treatment success rates reported in a systematic review and meta-analytical study (62%) [46] of those in Kelantan, Malaysia (57.1%) [47]; Morocco (53.5%) [48]; India (38%) [49]. This study’s TSR consisted of 32 (31.7%) patients who were declared cured, and 42 (41.6%) who had completed their treatment. Of the 27 (26.7%) patients with unsuccessful outcomes, 10 (9.9%) died, 8 (7.9%) were LTFU, and 8 (7.9%) were transferred out while 1 (1%) patient had treatment failure. There was no record of a treatment default in this study. The mortality rate (9.9%) in this study is comparable to that found in the study in Jos Nigeria [36] but lower than that reported in Zimbabwe, 26.4% [30]; Sierra Leone, 20.5% [38]; Uganda, 18.4% [43]; and Zambia, 16.7% [50]. The disparity in treatment outcomes between these studies may be due to differences in the study population’s age, gender, and disease severity, the existence of comorbid conditions, tobacco use, the drug resistance pattern, social determinants of health, and socioeconomic characteristics [51]. Another difficulty encountered in the TB control program is the category of patients that are lost to follow up (LTFU). This study recorded that 7.9% of patients were LTFU. Contrary to ours, previous studies in Durban South Africa recorded a higher proportion, which was 22.3% [1]. Others reported a proportion of 3.3% in Sierra Leone [38], 8.6% in Uganda [45], and 16.7% in Zambia [28]. The availability of trained medical personnel for follow-up checks, regular home visits by the treatment coordinator, proper social support from a psychiatrist, and the supply of free medication together with appropriate counseling by the pharmacist may all be contributing factors to the variation in LTFU rates between studies [51].

Male smokers with HIV had more time on treatment than did non-smokers. Compared to HIV-positive individuals who do not smoke, TB patients with HIV who smoke are more likely to have a worse response to treatment due to the harm that smoking causes. Smoking generally can reduce a treatment’s ability to save a life. Smoking negatively impacts efforts to treat TB-HIV co-infected patients and is linked to tuberculous infection complication, TB illness, and poor anti-tuberculosis treatment outcomes. The duration of treatment for HIV-negative or -positive patients and non-smokers decreased with age. The prevalence of smokers among TB patients in this study (34.7%) is lower than that reported by Lim et al. [52] and Khan et al. [51] who found prevalence rates of 46.4% and 46.2%, respectively, for smokers who were TB patients. Smoking is a risk factor for developing tuberculosis and is linked to unsuccessful treatment [51]. According to the 2014 US Surgeon General’s report on smoking, there is a causal link between smoking and an elevated risk of death and tuberculosis disease [53]. Wang et al. [54] in his study reported that smokers had higher chances of adverse outcomes, delayed smear or culture conversion, and treatment loss due to follow up as opposed to non-smokers.

## 5. Conclusions

The current study’s rate of successful TB treatment outcomes fell short of the 90% criterion set by WHO. The results of this study provide essential information for planning and executing TB and HIV activities as well as for modifying health services to meet the needs of diverse age groups and risk groups. Improving the treatment outcomes for TB patients requires early TB detection, the prompt commencement of effective anti-TB drug therapy, enhanced strategies for HIV prevention, and intensified health education efforts. The findings are highly recommended to be considered by policy makers to improve the policies in place.

## Figures and Tables

**Table 1:** Characteristics of the study participants in relation to treatment outcomes.

Patient Characteristics		Successful TB Treatment Outcome (N = 281) Ave No. (%)	Unsuccessful TB Treatment Outcome (N = 146) Ave No. (%)	*p*-Value
Age (years)		38.19 (14.96)	36.3 (15.4)	0.23
Gender (M and F)		130 (46.3)	59 (40.4)	0.25
Period between TSD and TED		326.5 (95.3)	186 (82.6)	<0.001
Previous drug history	New	160 (56.9)	63 (43.2)	<0.001
	PT1	110 (39.1)	57 (39.0)	
	PT2	11 (3.9)	25 (17.1)	
	UNK	0 (0.0)	1 (0.7)	
Type of resistance				
	Mono-resistance	130 (46.3)	71 (48.6)	0.64
	Poly-resistance	151 (53.7)	75 (51.4)	
Type of TB drug resistance				
	RR	131 (46.6)	74 (50.7)	0.012
	MDR	130 (46.3)	51 (34.9)	
	Pre-XDR	7 (2.5)	12 (8.2)	
	XDR	8 (2.8)	8 (5.5)	
	INH-R	5 (1.8)	1 (0.7)	
HIV Status				
	Positive	172 (61.2)	97 (66.4)	0.186
	Negative	109 (38.8)	49 (33.6)	
Age category				
	≤21	27 (9.6)	23 (15.8)	0.32
	>21:≤40	151 (53.7)	73 (50.0)	
	>40:≤55	56 (19.9)	32 (21.9)	
	>55:≤75	40 (14.2)	17 (11.6)	
	>75:≤90	5 (1.8)	1 (0.7)	

Data are presented as means (SD) for continuous measures, and n (%) for categorical measures. TSD—treatment start date; TED—treatment end date; PT1—previously treated with first-line drugs; PT2—previously treated with second-line drugs; UNK—unknown; RR—Rifampicin resistance; MDR—multidrug-resistant; XDR—extremely drug-resistant; INH-R—isoniazid-resistant.

**Table 2:** Association of treatment outcome with period between start and end of treatment and previous drug history.

TB Treatment Outcome	Odds Ratio (95% CI)	Std Error	Z	*p* > |z|
Treatment days	0.984 (0.981–0.987)	0.002	−10.22	0.000
Drug history	1.842 (1.232–2.753)	0.378	2.98	0.003
_cons	12.629 (4.651–34.289)	6.436	4.98	0.000

**Table 3:** (**a**) Addition of the type of TB drug resistance in the model. (**b**) Treatment outcome association with all variables.

(a)				
**TB Treatment Outcome**	**Odds Ratio (95% CI)**	**Std Error**	**Z**	***p* > |z|**
Treatment days	0.983 (0.980–0.987)	0.002	−10.24	0.0000
Drug history	1.841 (1.228–2.759)	0.380	2.96	0.003
Drug resistance	1.339 (0.984–1.823)	0.211	1.86	0.064
_cons	8.465 (2.893–24.770)	4.637	3.90	0.000
**(b)**				
**TB Treatment Outcome**	**Odds Ratio (95% CI)**	**Std Error**	**Z**	***p *** **> |z|**
Treatment days	0.984 (0.980–0.986)	0.002	−10.27	0.0000
HIV status	1.415 (0.787–2.544)	0.424	1.16	0.246
Drug resistance	1.246 (0.825–1.880)	0.262	1.04	0.296
Resistance type	1.266 (0.634–2.529)	0.447	0.67	0.503
Drug history	1.984 (1.296–3.036)	0.431	3.15	0.002
Age	1.025 (0.974–1.079)	0.027	0.95	0.340
Gender	0.708 (0.410–1.223)	0.198	−1.24	0.126
Age category	0.963 (0.918–1.011)	0.024	−1.53	0.126
_cons	11.109 (1.934–63.8111)	9.991	2.70	

**Table 4:** Socio-demographics of TB patients who were followed up.

Variable		Frequency (N)	Percentage (%)
Age	16–35 years	52	51.5
	36–60 years	40	39.6
	≥60 years	9	8.9
Sex	Male	65	64.4
	Female	36	35.6
HIV status	Positive	67	66.3
	Negative	34	33.7
Social History	Smoker	35	34.7
	Non-smoker	66	65.3

## Data Availability

Data can be requested from the corresponding author.
